# Supplementary material for: QED radiative corrections for accelerator neutrinos
Source: Nat Commun. 2022 Sep 8;13:5286. doi: 10.1038/s41467-022-32974-x (PMC9458660; doi:10.1038/s41467-022-32974-x)
Supplement: Supplementary file 1 — Description of Additional Supplementary Files [file 41467_2022_32974_MOESM1_ESM.pdf]

Supplementary Code 1: The script reproduces all plots and results in the paper. The incoming neutrino energy is 2 GeV, while the soft-photon energy cutoff is 10 MeV, and we consider electromagnetic jet of 10^0 size around the electron direction. For this calculation, we exploit the z-expansion parameterization of the nucleon vector form factors and corresponding uncertainties from Phys. Rev. D 102, 074012 (2020) and the axial form factor from Phys. Rev. D 93, 113015 (2016). For virtual contributions, we express all invariant amplitudes of (anti)neutrino-nucleon scattering in terms of the standard one-loop integrals. We take one-loop master integrals from the LoopTools package. For the contribution of real photons, we have performed the straightforward phase-space integration with corresponding phase-space cuts. The script combines virtual and real corrections and produces tables for all figures in the paper.
